# Editorial: Transparent machine learning in bio-medicine

**DOI:** 10.3389/fbinf.2023.1264803

**Published:** 2023-08-04

**Authors:** Juan G. Diaz Ochoa, André Marquardt

**Affiliations:** ^1^ PERMEDIQ GmbH, Wang, Germany; ^2^ QUIBIQ GmbH, Stuttgart, Germany; ^3^ Department of Pathology, Klinikum Stuttgart, Stuttgart, Germany

**Keywords:** machine learing, deep learning, artificial intelligence, transparence, explainability, privacy, safety, ethics

Current debates about AI risks and the possibility of humanity being taken over by a conscious artificial intelligence (AI) have a poor evidential base and are helping to neglect real existing problems and risks of AI technologies^1^. One of these problems is, for example, the fact that machine learning (ML) methods are essentially statistical models relying on complex datasets. Blind decisions in medicine based on non-transparent (but well validated) statistical methods can be dangerous when such models are not transparent enough to account for patient variability or the divergence of real-world data cohorts compared to training data.

However, it is expected that ML applications in medicine will expand at a compound annual growth rate of 37.5% from 2023 to 2030. Such high expectations set social and economic pressure to approve, standardize, and regulate ML methods in the bio-medical field^2^.

While Deep Learning (DL) methods have proven their value in precision oncology applications, they are difficult to implement in clinical routine care due to the common uninterpretable black-box character of these models. In their comprehensive review, “Opportunities and challenges in interpretable deep learning for drug sensitivity prediction of cancer cells”, Samal et al. are not only giving insights into eight different models of interpretable DL for the given task, but also introduce the three main different possible strategies of it: probing, perturbation, and surrogation. Focusing solely on the probing strategy, they introduce the three different classes of model probing—embeddings, gradients, and weights—and the different interpretation levels—global, semi-global, local, *ad hoc*, and *post hoc*. Determined by the input information and the chosen model the interpretability of an Artificial Neural Network (ANN) is set to a certain extent, leading to the conclusion that the reviewed methods are still not satisfactory enough. However, the authors observe, that the embedding-based method is the most promising of the three probing methods because it avoids the pitfalls associated with gradients and weights.

Not only the model’s transparency in data and architecture, but also the customer’s mental state and the intended use of the model must be taken into consideration (Jacob, 2023). This issue is addressed by Ochoa et al. addressed in their work, “Bayesian logical neural networks for human-centered applications in medicine”, posing the fundamental question of the trade-off between the performance and transparency of a model (Ochoa et al.). In their work, Ochoa et al. combine Logical Neural Networks (LONNs) with Bayesian Neural Networks (BNNs) creating a new model - BaLONNs - combining the characteristics of both approaches. The introduction of individual invariant layers with fixed weights and biases with squashing functions allows *a posteriori* interpretation of the DL layers as logical gates, ultimately resulting in increased model transparency. Furthermore, Bayesian-Modeling techniques allow the construction of models that inform the customer both of the accuracy and the remaining uncertainty of the predictions made by the model.

While transparency is considered central for an ethical implementation of ML, in recent years it has become clear that there is a conflict between model transparency and data privacy (Grant and Wischik, 2020) Considering that both explainable and transparent modelling methods can provide access to critical private data, e.g., patient data, Lucieri et al., in their work “Translating Theory into Practice: Assessing the Privacy Implications of Concept-based Explanations for Biomedical AI” (Lucieri et al.), question the problem that model-explainability possesses for training dataset privacy. Explainability is providing a clear relation between groups of features in input data and model-outputs (training-explainability). Thus, by reverse modelling it should be possible to infer characteristics in input-data from output predictions, i.e., use output predictions to train models in inferring characteristics and patterns in input data that could compromise privacy. The member inference attack is a type of reverse modelling that can be considered a privacy attack. One way to solve this problem is by implementing data-privacy techniques in the original training data. Nevertheless, Lucieri et al. contend that data-privacy can have the unintended consequence of enabling privacy attacks to succeed, and that an appropriate balance must be struck between concept-based explainability and privacy (Lucieri et al.).

Transparency and explainability refers not only to model or data transparency, but also to the potential risk that inaccurate model predictions pose. Regarding this, Alipanahi et al. provide a review, with the title “CRISPR genome editing using computational approaches: A survey”, about the role and implications of deep learning to mitigate off-target effects in CRISPR/Cas9 (Alipanahi et al.). CRISPR research have been focused on two fundamental questions: how to calculate potential targets of gRNAs and how to be confident about the accuracy of CRISPR edits. Most research in CRISPR area focus on increasing cleavage activity and efficiency, leading to more undesired off-target cleavage. Therefore, a balance between these two criteria must be maintained by designing successful CRISPR gRNA and choosing an appropriate Cas protein. To this end, several gRNAs must be evaluated using multiple models and databases to select the best one for their experiments. Previous successes of DL architectures motivated the use of DL platforms as the best solution for predicting off-target effects. However, their accuracy depends on the quality and amount of available data, which can compromise the quality of the results obtained with these platforms. Transparency in the training data is thus relevant for the quality of predictions made in this field.

The most significant conclusion to be drawn from this article Research Topic are, first, that techniques aiming to get more model transparency for clinical applications are still not satisfactory enough, and second, that the right balance between explainability and validation is required, and that validation metrics are not the sole determinant of which model to select. Furthermore, to ensure safe ML, data and model transparency, including structural transparency, are vital components. Thus, transparency, privacy and reliability must be all fine-tuned, perhaps not from a universal perspective, but rather from a local one.

## Footnotes

1 Not emergent AI consciousness, but the current infrastructure required for AI has become very dangerous. See, e.g., the interview to Meredith Wittaker: https://www.republik.ch/2023/07/05/wer-dem-ki-hype-verfaellt-staerkt-die-macht-der-big-tech-chefs?utm_source=pocket-newtab-global-de-DE

2
https://eur-lex.europa.eu/eli/reg/2016/679/2016-05-04
